# Is Opium Addiction a Risk Factor for Bone Loss?

**Published:** 2011-07-01

**Authors:** M H Gozashti, A Shahesmaeili, N Amini Zadeh

**Affiliations:** 1 Kerman Neuroscience Research Center, Kerman University of Medical Sciences, Kerman, Iran; 2 Department of Endocrinology,Kerman University of Medical Sciences, Kerman, Iran; 3 Physiology Research Center, Kerman University of Medical Sciences, Kerman, Iran; 4 Shahid Beheshti University of Medical Sciences, Tehran, Iran

**Keywords:** Bone mineral density, Bone loss, Opium dependency, osteopenia

## Abstract

**Background:**

Drug abuse is one of the most important public health problems worldwide as in Iran. The aim of present study was to determine whether opium addiction can affect bone mineral density or not.

**Methods:**

Fifty opium addicted and 50 non-addicted volunteer men aged between 25-45 were enrolled. The subjects with positive history of other osteoporosis risk factors were excluded. The vertebral bone density and potential confounders (age, cigarette smoking and body mass index) were measured in all subjects.

**Results:**

Twenty six percent of non-addicted vs. 56% of addicted subjects had vertebral osteopenia. According to adjusted ORs, addiction to opium (OR: 3.08, CI95% 1.20-7.92) and age (OR: 1.11 CI95% 1.03-1.20) were significantly related to vertebral bone loss.

**Conclusion:**

Opium addicted patients were more susceptible to bone loss than non-addicted individuals. So,early screening and conducting prevention programs should be taken into consideration for this high risk group.

## Introduction

Bone mineral density (BMD) is a good index for evaluating bone status and diagnosis of metabolic bone disorders. Osteoporosis and osteopenia are the most prevalent metabolic bone diseases that are characterized by low BMD.[1][2][3]Osteoporosis is an asymptomatic disease that predisposes the patients to bone fragility and fracture after minimal trauma or fall down.[3][4] It is a disturbing public health problem because of its consequent morbidity, mortality, medical costs and long term cares.[5][6]

Recent data shows that in Iran, the prevalence of osteoporosis and osteopenia in individuals aged 50 and above was 36.1% and 43.9% in women, and 24.5% and 70.8% in men, respectively. In addition, among subjects under 50 years, 49.6% of women and 59.6% of men have low bone mass.[7] According to the Endocrinology and Metabolism Research Center (EMRC) report, two million people in Iran are at the risk of fracture.

The annual cost of hip fractures in Iran is between 8,000,000 and 16,000,000 US $.[8] These data indicate that loss of bone density and its consequent complications are major public health problem in Iran as other developing countries. A study about the burden of osteoporosis in 2004, showed that Disability Adjusted Life Years (DALYs) attributable to osteoporosis is 36026 among Iranian population (18757 in men and 17270 in women).[2]

The main risk factors of decreased bone mineral density are: age, gender, life style, nutrition, hormonal changes and some medical conditions. It has been reported that 20-50% of bone mass changes are related to life style. Immobility, smoking, alcohol drinking and low calcium and vitamin D intake are the most important lifestyle factors that affect BMD.[9] So, the prevention programs that focus on the life style development and screening of high risk groups can play an important role in the early diagnosis, treatment and decreasing of morbidity and complications of the disease.

Opium abuse is a major public health problem worldwide as in our country. It is considered as the most common type of drug abuse in Iran.[10] It is estimated that about 5-9% of Iranian population are addicted to opium.[11][12] The effect of opium addiction on the bone mineral density is not clear. Only one study in 1993 showed that chronic heroin abuse is associated with altered bone metabolism and reduced trabecular bone mass.[13] Other studies indicate that chronic consumption of opiates can induce hypogonadim and increase serum level of prolactin.[14][15][16][17] Both hypogonadism and hyperprolactinemia may lead to osteoporosis.

So, considering the high prevalence of opium addiction in Iran and the lack of data that reveal the effect of opium abuse on the BMD, based on review of literature, we examined the relationship between chronic opiate abuse and bone mass. The confounding roles of the other potential risk factors were also considered.

## Materials and Methods

In this case-control study, we enrolled 50 opium addicted and 50 non-addicted volunteer men aged between 25-45 years. All patients were interviewed based on the DSM-IV criteria for opium dependency. The morphine urine test was conducted in the control group. To confirm the inclusion of participants in the study, subjects with following diseases or conditions were excluded before densitometry: known history or evidence of rheumatoid arthritis, thyroid, parathyroid or adrenal disease, hepatic or renal failure, metabolic bone disease, type I diabetes mellitus, sterility, malabsorption, immobility for more than one week and alcoholism. All remaining subjects signed an informed consent which explained all aspects of study and asked for the permission of bone densitometry test. Body mass index (BMI) was measured in all subjects.

BMD was measured once at the lumbar spine (L2- L4) with dual X-ray absorptiometry using Lunar DPX densitometers (Lunar 7164, GE, and Madison, WI). The procedure was carried out by a trained operator according to the manufacturer’s instruction. Machine calibration was done on a daily basis. Daily and weekly quality assurance tests were performed as recommended by the DXA machine manufacturers. For classification of bone marrow densitometry results, we used World Health Organization criteria .According to the criteria, the T- score between -1 to - 2.5 standard deviation was considered as osteopenia and T-score≤-2.5 standard deviation was regarded as osteoporosis. The normal BMD was defined as having T-score≥-1 standard deviation.[18]

The data were entered and analyzed by SPSS17 software (Version 15, Chicago, IL, USA). In order to explore the relationship between opium consumption (as the predictor) and the bone marrow density, univariate and multiple logistic regression models were applied. According to above definition, osteopenia and osteoporosis were categorized as bone loss and entered as dependent variable in the model. Other potential confounders (age, cigarette smoking and body mass index) were also entered in the model as predictor variables. The crude and adjusted OR was reported. P values less than 0.05 were considered as significant level.

## Results

The mean age of addicted and non-addicted groups was 36.00±6.54 and 35.38±6.7 years respectively. There was not any significant difference between the age and history of cigarette smoking in two groups but the mean of BMI in addicted group was significantly lower than that of non-addicted ones (p=0.002, Table 1).

According to WHO criteria, 26% of non-addicted and 56% of addicted individuals had osteopenia which shows the difference between the two groups to be statistically significant (p=0.011). None of the subjects had osteoporosis. The mean lumbar measurement in opium addicted group was statistically lower than non addicted ones.

To study the confounding effects of cigarette smoking, BMI and age on opium addiction and bone density relation, the multivariate analysis was performed. According to adjusted ORs, addiction to opium (OR=3.08, CI95% 1.20-7.92) and age (OR=1.11 CI95% 1.03-1.20) were significantly related to lumbar bone loss (Table 2).

**Table 1:** The comparison of studied variables and bone status in opium addicted and contol groups

**Variable**	**Opium addicted N=50**	**Non addicted N=50**	**P value**
Age Mean±SD	36.00±6.54	35.38±6.7	0.64
BMI Mean±sD	21.96±3.46	24.27±3.85	0.002
Cigarrette smoking No. (%)	37 (77.1)	34 (61)	0.31
Vertebral BMD frequency (%)			
Normal	22 (44)	37 (74)	0.002
Osteopenia	28 (56)	13 (26)	
Lumbar measurement Mean±SD	0.92±0.12	1.01±0.12	0.001

**Table 2:** Univariate and Multivariable analysis of the studied variables that predicting the bone mineral density

**Variables**	**Crude Analysis**			**Adjusted Analysis**		
	**OR**	**CI 95%**	**P value**	**OR**	**CI 95%**	**P value**
Opium addiction	3.62	1.55-8.41	0.003	3.08	1.20-7.92	0.019
Cigarette smoking	1.24	0.50-3.09	0.63	0.83	0.29-2.40	0.74
Age	1.096	1.02-1.17	0.006	1.11	1.03-1.20	0.003
BMI	0.87	0.78-0.98	0.023	0.89	0.78-1.02	0.121

## Discussion

The findings of this study indicate that opium addicted individuals are more susceptible to loss of lumbar BMD than non-addicted ones. There are some researches about the possible mechanisms of opium osteoporosis. Pedrazzoni et al. in 1993 revealed that the vertebral bone trabecular mass and bone density in individuals with the history of chronic heroine abuse are significantly lower than control group. They also showed an increased level of serum ionized calcium and urine hydroxyproline and decreased level of serum LH and PTH in heroin addicted group compared to controls13 Opium can interact with bone metabolism in different ways: some researches established that opium abuse can suppress hypotalamohypophysio- gonadal axis and consequently decreases the level of gonadal hormones.[13],[19][20][21] It is well known that chronic hypogonadism is a prominent cause of osteoporosis in both sexes.[19][20][21] Hejazian et al. in 2007 revealed that the level of serum testosterone, LH and FSH hormones significantly decrease in opium addicted group as compared with the control group.19 In 2009, Fraser et al. found that in patients who received long term opiates to relieve the pain, there is a much higher prevalence of hypogonadism in men than in women (75% vs. 21%). In their study, osteopenia was reported in 50% of men and 21% of women which was statistically significant.[14]

Moshtaghi-Kashanian et al. in 2006 showed an increased level of prolactin in concurrent opium and cigarette smokers in comparison with cigarette smokers alone (86.96% vs. 41.65%).[15] Other researches also reached the same results.[16][22][23] Hyperprolactinemia is one of the well known risk factors for developing bone loss that is another explanation for increased risk of bone loss and bone fracture in opium addicted persons. In addition to indirect effects of opium, this substance may also contribute to lowered bone mineral density and increased fracture risk by directly interfering with bone formation.[19] Inhibition of human osteoblastic tissue cultures growth, decrease in serum osteocalcin levels and finally inhibition of osteocalcin production by osteoblast tissue cultures are some direct effects of opium on bone formation.[19]

Opium consumption is usually accompanied by specific risky life styles such as cigarette smoking, low physical activity and poor nutrition. Poor nutritional status among them who are addicted to opium is a result of economical problems and loss of appetite. Opium addiction can lead to low dietary intake of calcium and phosphor and indirectly makes the individual more prone to bone loss.

Although in our study the cigarette smoking was not related to bone loss but there is a growing body of evidence that suggest cigarette smoking is a risk factor for osteoporosis, but the nature of this relationship is not clear.[24] Some studies indicate that cigarette smoking is a risk factor for osteoporosis and bone loss.[25][26][27][28] But other studies have found no obvious evidence about the relationship between cigarette smoking and loss of bone density.[19][29][30]

This difference among available data can be due to variation in study design, gender of subjects, sample size and considering the probable confounding factors (such as BMI).

We can point to these aspects among the most important strengths of our study: First, we considered some well known factors and medical conditions that may influence bone mass as exclusion criteria. Second, we have carried out our study on male samples to set aside female hormonal changes that may affect bone mass. Third, we chose the individuals aged between 25-45 years to avoid a mix up with age related bone loss that often occurs above 50 years and finally multivariate analysis considered the effect of main potential confounders.

The main limitation of our study was that we did not measure the level of gonadal hormones and some bone markers such as urine hydroxyproline, calcium, phosphor and alkaline phosphatase in our samples. We strongly recommend conducting more precise studies to measure these bone markers in sera of the subjects. Our results suggest that opium addicted patients are more susceptible to bone loss than non-addicted individuals. So, early screening and conducting prevention programs should be taken into consideration for this high risk group. Without any doubt, further studies to explore responsible mechanisms are needed.
